# Benzyl­tris­[2-(di­benzyl­amino)­eth­yl]ammonium iodide

**DOI:** 10.1107/S1600536813031607

**Published:** 2013-12-04

**Authors:** Mollie J. Bello, Sarah E. Brady, Lev N. Zakharov, David R. Tyler

**Affiliations:** a Department of Chemistry, 1253 University of Oregon, Eugene, Oregon 97403-1253, USA; b CAMCOR, University of Oregon, 1443 E. 13th Avenue, Eugene, Oregon 97403, USA

## Abstract

In the title quaternary ammonium salt, C_55_H_61_N_4_^+^·I^−^, all three *N*,*N*-di­benzyl­ethanamine, –(CH_2_)_2_N(CH_2_C_6_H_5_)_2_, groups have different conformations. The N—C—C—N torsion angles are significantly different [89.86 (13), 162.61 (10) and 175.70 (10)°] and the dihedral angles between the phenyl rings in these groups are different as well [58.21 (4), 43.73 (4) and 76.72 (5)°]. In the crystal, the I^−^ anions fill empty spaces between the bulky cations. The cations and anions are linked by weak C—H⋯I inter­actions, forming a chain along [110].

## Related literature   

For related ligand structures, see: Farrell *et al.* (2003 ▶). For the application of similar ligands coordinating to copper in the catalysis of atom-transfer radical polymerization or click reactions, see: Barré *et al.* (2004 ▶); Candelon *et al.* (2008 ▶); Liang *et al.* (2011 ▶); Brady & Tyler (2012 ▶).
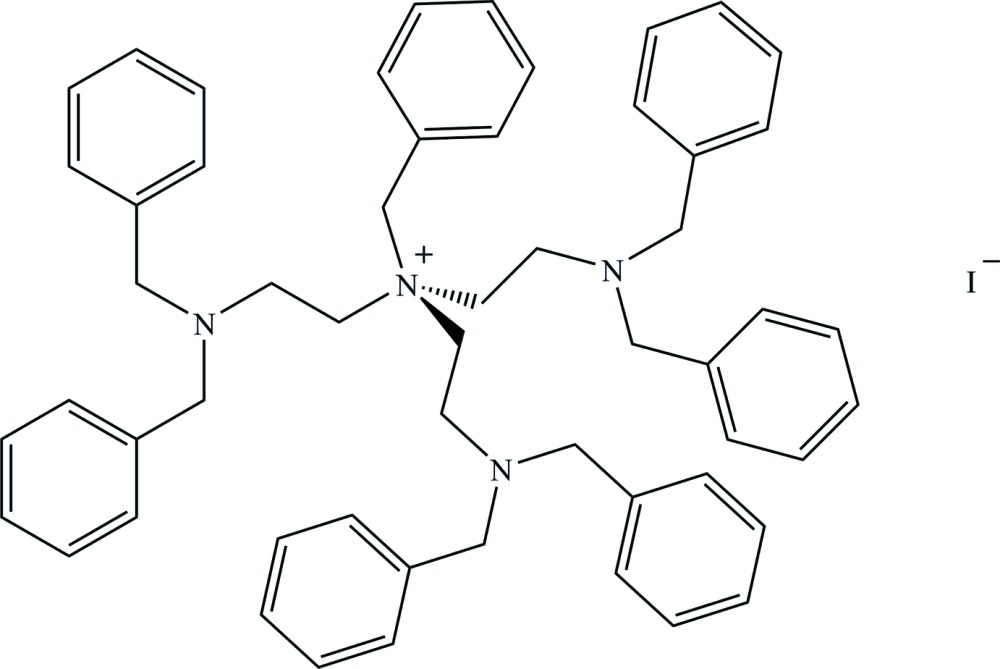


## Experimental   

### 

#### Crystal data  

C_55_H_61_N_4_^+^·I^−^
*M* *_r_* = 904.98
Triclinic, 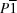

*a* = 11.1254 (10) Å
*b* = 14.3871 (13) Å
*c* = 15.9525 (15) Å
α = 94.491 (3)°
β = 101.972 (3)°
γ = 106.229 (3)°
*V* = 2372.9 (4) Å^3^
*Z* = 2
Mo *K*α radiation
μ = 0.72 mm^−1^
*T* = 100 K
0.25 × 0.24 × 0.11 mm

#### Data collection  

Bruker APEXII CCD diffractometer
Absorption correction: multi-scan (*SADABS*; Bruker, 2000 ▶) *T*_min_ = 0.842, *T*_max_ = 0.926
53881 measured reflections
10377 independent reflections
9802 reflections with *I* > 2σ(*I*)
*R*_int_ = 0.030

#### Refinement  

*R*[*F* ^2^ > 2σ(*F* ^2^)] = 0.020
*wR*(*F* ^2^) = 0.049
*S* = 1.04
10377 reflections
542 parameters
H-atom parameters constrained
Δρ_max_ = 0.38 e Å^−3^
Δρ_min_ = −0.42 e Å^−3^

### 

Data collection: *APEX2* (Bruker, 2008 ▶); cell refinement: *SAINT* (Bruker, 2000 ▶); data reduction: *SAINT*; program(s) used to solve structure: *SHELXTL* (Sheldrick, 2008 ▶); program(s) used to refine structure: *SHELXTL*; molecular graphics: *SHELXTL*; software used to prepare material for publication: *SHELXTL*.

## Supplementary Material

Crystal structure: contains datablock(s) I. DOI: 10.1107/S1600536813031607/is5322sup1.cif

Structure factors: contains datablock(s) I. DOI: 10.1107/S1600536813031607/is5322Isup2.hkl

Supporting information file. DOI: 10.1107/S1600536813031607/is5322Isup3.cdx

Supporting information file. DOI: 10.1107/S1600536813031607/is5322Isup4.cml

Additional supporting information:  crystallographic information; 3D view; checkCIF report

## Figures and Tables

**Table 1:** Hydrogen-bond geometry (Å, °)

*D*—H⋯*A*	*D*—H	H⋯*A*	*D*⋯*A*	*D*—H⋯*A*
C39—H39*A*⋯I1^i^	0.95	3.01	3.893 (2)	154
C49—H49*B*⋯I1^ii^	0.99	2.86	3.8187 (13)	162

Symmetry codes: (i) 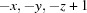; (ii) 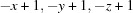.

**Fig. 1.**
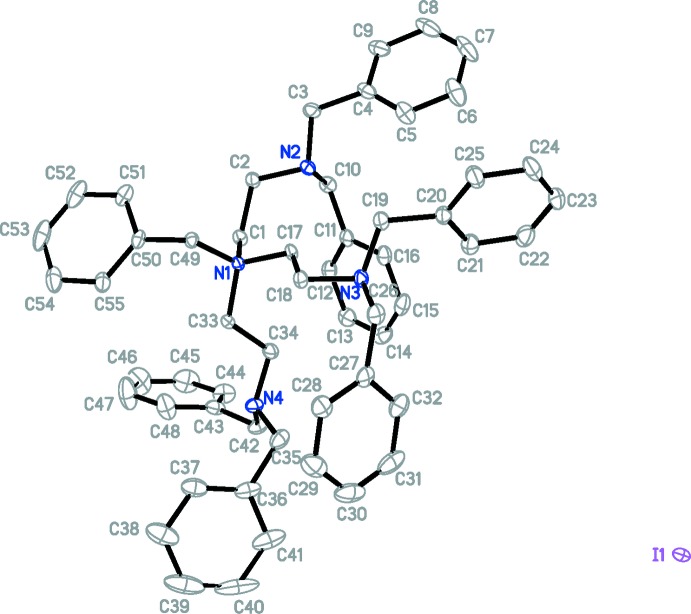
The molecular structure of the title compound, with 50% probability displacement ellipsoids and the atom-numbering scheme. H atoms are omitted for clarity.

## supplementary crystallographic information

### 1. Comment

Work in our laboratory has focused on the preparation of nonlinear polymers containing metal-metal bonds. Most recently, synthetic methods using the Huisgen 1,3-dipolar cycloaddition click reaction between 1,3,5-triethynylbenzene and an azide-functionalized molybdenum dimer, [(*η*^5^-C_5_H_4_(CH_2_)_3_N_3_)Mo(CO)_3_]_2_, have been used. It was concluded that 1,3,5-triethynylbenzene could not be coupled to azide functionalized molecules under click reaction conditions. 1,3,5-Triethynylbenzene reacted with Cp*Ru(COD)Cl instead of clicking, and Cu(IMes)Cl was too sterically bulky to catalyze three cycloadditions (Brady & Tyler, 2012). However, similar results demonstrated that [Cu(I)tren(C_18_H_37_)_6_]Br can catalyze the preparation of dendrimers by the Huisgen click reaction (Candelon *et al.*, 2008; Barré *et al.*, 2004). Dendritic analogues of the catalyst have also been prepared and used to synthesize second generation dendrimers with 54 terminal groups (Liang *et al.*, 2011). These examples suggest that sterics do not effect the catalytic activity of [Cu(I)tren(C_18_H_37_)_6_]Br. This warranted the examination of [Cu(I)tren(C_18_H_37_)_6_]Br as a catalyst to prepare a star polymer with 1,3,5-triethynylbenzene.

The title compound (1) was prepared following literature preparations for [Cu(I)tren(C_18_H_37_)_6_]Br. Unfortunately, 1 did not coordinate to copper so it was not examined as a click chemistry catalyst.

### 2. Experimental

The title compound was prepared based on a literature procedure in which three molar equivalents of bromomethylbenzene were used (Farrell *et al.*, 2003). As reported here, six molar equivalents of bromomethylbenzene were used. Tren (0.15 g, 1.04 mmol) was dissolved in acetonitrile (20 ml) and degassed. K_2_CO_3_ (0.55 g, 4.16 mmol) and KI (0.66 g, 4.24 mmol) were added under nitrogen. A degassed solution of bromomethylbenzene (1.08 g, 6.30 mmol) in acetonitrile (20 ml) was cannulated into the solution of deprotonated tren. The reaction solution was refluxed at 95 °C for 16 h after which time the reaction mixture was still cloudy and white. The solution was cooled to 0 °C in an ice bath and the solvent was removed *in vacuo*. The resulting white powder was dissolved in dichloromethane and washed with water, Na_2_S_2_O_3_, and water again. The solvent was removed *in vacuo* to yield a yellow oil. The oil was dissolved in ethyl acetate (30 ml) and heptane (15 ml) and slowly heated. Upon heating, a white powder precipitated. Based on the ^1^H NMR spectrum and the melting point (found: 31–125 °C, reported 50–53 °C) the product is not pure. X-ray quality crystals were prepared by slow evaporation from an ethanol solution.

### 3. Refinement

H atoms were positioned geometrically (C—H = 0.95 or 0.99 Å) and refined in a rigid group model, with *U*_iso_(H) = 1.2*U*_eq_(C).

### Crystal data

**Table:**

C_55_H_61_N_4_^+^·I^−^	*Z* = 2
*M**_r_* = 904.98	*F*(000) = 944
Triclinic, *P*1	*D*_x_ = 1.267 Mg m^−^^3^
Hall symbol: -P 1	Mo *K*α radiation, λ = 0.71073 Å
*a* = 11.1254 (10) Å	Cell parameters from 9619 reflections
*b* = 14.3871 (13) Å	θ = 2.6–36.2°
*c* = 15.9525 (15) Å	µ = 0.72 mm^−^^1^
α = 94.491 (3)°	*T* = 100 K
β = 101.972 (3)°	Prizm, colourless
γ = 106.229 (3)°	0.25 × 0.24 × 0.11 mm
*V* = 2372.9 (4) Å^3^	

### Data collection

**Table:**

Bruker APEXII CCD diffractometer	10377 independent reflections
Radiation source: fine-focus sealed tube	9802 reflections with *I* > 2σ(*I*)
Triumph mirror monochromator	*R*_int_ = 0.030
φ and ω scans	θ_max_ = 27.0°, θ_min_ = 1.5°
Absorption correction: multi-scan (*SADABS*; Bruker, 2000)	*h* = −14→14
*T*_min_ = 0.842, *T*_max_ = 0.926	*k* = −18→16
53881 measured reflections	*l* = −20→20

### Refinement

**Table:**

Refinement on *F*^2^	Secondary atom site location: difference Fourier map
Least-squares matrix: full	Hydrogen site location: inferred from neighbouring sites
*R*[*F*^2^ > 2σ(*F*^2^)] = 0.020	H-atom parameters constrained
*wR*(*F*^2^) = 0.049	*w* = 1/[σ^2^(*F*_o_^2^) + (0.0153*P*)^2^ + 1.324*P*] where *P* = (*F*_o_^2^ + 2*F*_c_^2^)/3
*S* = 1.04	(Δ/σ)_max_ = 0.002
10377 reflections	Δρ_max_ = 0.38 e Å^−^^3^
542 parameters	Δρ_min_ = −0.42 e Å^−^^3^
0 restraints	Extinction correction: *SHELXTL* (Sheldrick, 2008), Fc^*^=kFc[1+0.001xFc^2^λ^3^/sin(2θ)]^-1/4^
Primary atom site location: structure-invariant direct methods	Extinction coefficient: 0.00066 (14)

### Special details

**Table:**

Geometry. All e.s.d.'s (except the e.s.d. in the dihedral angle between two l.s. planes) are estimated using the full covariance matrix. The cell e.s.d.'s are taken into account individually in the estimation of e.s.d.'s in distances, angles and torsion angles; correlations between e.s.d.'s in cell parameters are only used when they are defined by crystal symmetry. An approximate (isotropic) treatment of cell e.s.d.'s is used for estimating e.s.d.'s involving l.s. planes.
Refinement. Refinement of *F*^2^ against ALL reflections. The weighted *R*-factor *wR* and goodness of fit *S* are based on *F*^2^, conventional *R*-factors *R* are based on *F*, with *F* set to zero for negative *F*^2^. The threshold expression of *F*^2^ > σ(*F*^2^) is used only for calculating *R*-factors(gt) *etc*. and is not relevant to the choice of reflections for refinement. *R*-factors based on *F*^2^ are statistically about twice as large as those based on *F*, and *R*- factors based on ALL data will be even larger.

### Fractional atomic coordinates and isotropic or equivalent isotropic displacement parameters (Å^2^)

**Table:**

	*x*	*y*	*z*	*U*_iso_*/*U*_eq_	
I1	0.258461 (8)	0.304462 (6)	0.946780 (6)	0.01859 (3)	
N1	0.65830 (9)	0.40260 (7)	0.14758 (6)	0.0115 (2)	
N2	0.92688 (10)	0.57153 (8)	0.27511 (7)	0.0148 (2)	
N3	0.47013 (10)	0.54906 (8)	0.24366 (7)	0.0153 (2)	
N4	0.46942 (10)	0.17927 (8)	0.22778 (7)	0.0174 (2)	
C1	0.79639 (11)	0.40571 (9)	0.18740 (8)	0.0141 (2)	
H1A	0.8182	0.3557	0.1524	0.017*	
H1B	0.8006	0.3872	0.2462	0.017*	
C2	0.89895 (12)	0.50425 (9)	0.19438 (8)	0.0157 (2)	
H2A	0.9800	0.4917	0.1882	0.019*	
H2B	0.8708	0.5373	0.1452	0.019*	
C3	1.01512 (12)	0.66504 (10)	0.26363 (9)	0.0194 (3)	
H3A	0.9779	0.6844	0.2083	0.023*	
H3B	1.0982	0.6549	0.2593	0.023*	
C4	1.04092 (13)	0.74770 (10)	0.33570 (9)	0.0208 (3)	
C5	0.94103 (15)	0.76527 (11)	0.36803 (10)	0.0282 (3)	
H5A	0.8557	0.7224	0.3468	0.034*	
C6	0.96470 (19)	0.84485 (12)	0.43104 (12)	0.0383 (4)	
H6A	0.8959	0.8557	0.4531	0.046*	
C7	1.08893 (19)	0.90862 (11)	0.46183 (11)	0.0397 (4)	
H7A	1.1053	0.9633	0.5046	0.048*	
C8	1.18838 (18)	0.89196 (11)	0.42988 (11)	0.0358 (4)	
H8A	1.2733	0.9356	0.4505	0.043*	
C9	1.16503 (15)	0.81164 (11)	0.36767 (9)	0.0266 (3)	
H9A	1.2344	0.8003	0.3468	0.032*	
C10	0.98820 (12)	0.53424 (10)	0.35122 (9)	0.0178 (3)	
H10A	1.0436	0.5907	0.3950	0.021*	
H10B	1.0447	0.4983	0.3331	0.021*	
C11	0.89454 (12)	0.46742 (10)	0.39318 (8)	0.0166 (2)	
C12	0.90450 (13)	0.37526 (10)	0.40752 (8)	0.0196 (3)	
H12A	0.9692	0.3535	0.3891	0.024*	
C13	0.82121 (14)	0.31463 (11)	0.44837 (9)	0.0235 (3)	
H13A	0.8297	0.2522	0.4579	0.028*	
C14	0.72586 (14)	0.34508 (12)	0.47524 (9)	0.0255 (3)	
H14A	0.6684	0.3036	0.5028	0.031*	
C15	0.71505 (14)	0.43675 (12)	0.46156 (9)	0.0254 (3)	
H15A	0.6499	0.4580	0.4799	0.030*	
C16	0.79869 (13)	0.49771 (11)	0.42131 (9)	0.0205 (3)	
H16A	0.7907	0.5605	0.4128	0.025*	
C17	0.62902 (11)	0.48370 (9)	0.19850 (8)	0.0134 (2)	
H17A	0.6886	0.5471	0.1926	0.016*	
H17B	0.6480	0.4758	0.2605	0.016*	
C18	0.49129 (12)	0.48881 (9)	0.17232 (8)	0.0152 (2)	
H18A	0.4789	0.5185	0.1185	0.018*	
H18B	0.4291	0.4223	0.1618	0.018*	
C19	0.54626 (13)	0.65255 (10)	0.25103 (9)	0.0191 (3)	
H19A	0.5050	0.6821	0.2036	0.023*	
H19B	0.6335	0.6559	0.2438	0.023*	
C20	0.55863 (12)	0.71169 (10)	0.33658 (9)	0.0187 (3)	
C21	0.57529 (13)	0.67301 (11)	0.41403 (9)	0.0227 (3)	
H21A	0.5734	0.6065	0.4131	0.027*	
C22	0.59459 (14)	0.73056 (12)	0.49277 (10)	0.0281 (3)	
H22A	0.6061	0.7033	0.5451	0.034*	
C23	0.59709 (16)	0.82747 (12)	0.49503 (10)	0.0328 (4)	
H23A	0.6109	0.8669	0.5488	0.039*	
C24	0.57942 (19)	0.86637 (12)	0.41876 (11)	0.0377 (4)	
H24A	0.5801	0.9326	0.4200	0.045*	
C25	0.56057 (16)	0.80891 (11)	0.33983 (10)	0.0290 (3)	
H25A	0.5489	0.8365	0.2877	0.035*	
C26	0.33205 (12)	0.54272 (10)	0.22862 (9)	0.0194 (3)	
H26A	0.3037	0.5604	0.1707	0.023*	
H26B	0.3220	0.5912	0.2721	0.023*	
C27	0.24552 (12)	0.44284 (10)	0.23376 (9)	0.0198 (3)	
C28	0.15843 (13)	0.38517 (11)	0.15984 (10)	0.0251 (3)	
H28A	0.1569	0.4075	0.1053	0.030*	
C29	0.07355 (15)	0.29513 (12)	0.16496 (12)	0.0349 (4)	
H29A	0.0149	0.2565	0.1140	0.042*	
C30	0.07459 (16)	0.26210 (12)	0.24384 (14)	0.0391 (4)	
H30A	0.0156	0.2013	0.2476	0.047*	
C31	0.16209 (16)	0.31805 (13)	0.31769 (12)	0.0355 (4)	
H31A	0.1639	0.2949	0.3720	0.043*	
C32	0.24727 (14)	0.40774 (12)	0.31282 (10)	0.0263 (3)	
H32A	0.3071	0.4454	0.3638	0.032*	
C33	0.57049 (11)	0.30202 (9)	0.15075 (8)	0.0133 (2)	
H33A	0.5978	0.2528	0.1189	0.016*	
H33B	0.4818	0.2975	0.1196	0.016*	
C34	0.56666 (13)	0.27480 (10)	0.24087 (8)	0.0194 (3)	
H34A	0.6519	0.2713	0.2716	0.023*	
H34B	0.5429	0.3240	0.2755	0.023*	
C35	0.34292 (14)	0.18551 (11)	0.23591 (10)	0.0237 (3)	
H35A	0.3258	0.2419	0.2094	0.028*	
H35B	0.3427	0.1961	0.2979	0.028*	
C36	0.23870 (13)	0.09302 (11)	0.19179 (11)	0.0254 (3)	
C37	0.21898 (14)	0.06601 (11)	0.10272 (11)	0.0285 (3)	
H37A	0.2703	0.1069	0.0712	0.034*	
C38	0.12591 (15)	−0.01933 (13)	0.05975 (14)	0.0415 (4)	
H38A	0.1143	−0.0370	−0.0007	0.050*	
C39	0.04972 (16)	−0.07896 (13)	0.10474 (18)	0.0511 (6)	
H39A	−0.0151	−0.1371	0.0751	0.061*	
C40	0.06773 (16)	−0.05418 (14)	0.19217 (18)	0.0540 (7)	
H40A	0.0157	−0.0957	0.2229	0.065*	
C41	0.16278 (15)	0.03243 (13)	0.23692 (14)	0.0389 (4)	
H41A	0.1748	0.0492	0.2975	0.047*	
C42	0.51333 (13)	0.10956 (10)	0.27861 (9)	0.0211 (3)	
H42A	0.4379	0.0545	0.2811	0.025*	
H42B	0.5570	0.1422	0.3385	0.025*	
C43	0.60544 (13)	0.07035 (10)	0.23936 (10)	0.0218 (3)	
C44	0.71526 (15)	0.05800 (12)	0.29130 (11)	0.0302 (3)	
H44A	0.7368	0.0789	0.3520	0.036*	
C45	0.79374 (16)	0.01507 (14)	0.25478 (13)	0.0412 (4)	
H45A	0.8684	0.0067	0.2907	0.049*	
C46	0.76366 (18)	−0.01528 (16)	0.16680 (15)	0.0492 (5)	
H46A	0.8160	−0.0461	0.1422	0.059*	
C47	0.65645 (19)	−0.00045 (17)	0.11451 (14)	0.0519 (6)	
H47A	0.6366	−0.0194	0.0536	0.062*	
C48	0.57798 (15)	0.04207 (13)	0.15092 (11)	0.0353 (4)	
H48A	0.5046	0.0518	0.1147	0.042*	
C49	0.64012 (12)	0.41892 (9)	0.05210 (8)	0.0136 (2)	
H49A	0.5471	0.4068	0.0266	0.016*	
H49B	0.6849	0.4883	0.0498	0.016*	
C50	0.68975 (13)	0.35503 (10)	−0.00260 (8)	0.0163 (2)	
C51	0.81668 (14)	0.38692 (12)	−0.01157 (9)	0.0239 (3)	
H51A	0.8725	0.4486	0.0183	0.029*	
C52	0.86189 (16)	0.32936 (14)	−0.06375 (10)	0.0332 (4)	
H52A	0.9488	0.3510	−0.0684	0.040*	
C53	0.78044 (18)	0.24072 (14)	−0.10873 (10)	0.0366 (4)	
H53A	0.8110	0.2017	−0.1450	0.044*	
C54	0.65424 (18)	0.20851 (12)	−0.10119 (10)	0.0320 (4)	
H54A	0.5986	0.1474	−0.1323	0.038*	
C55	0.60823 (14)	0.26518 (10)	−0.04828 (9)	0.0214 (3)	
H55A	0.5215	0.2427	−0.0433	0.026*	

### Atomic displacement parameters (Å^2^)

**Table:**

	*U*^11^	*U*^22^	*U*^33^	*U*^12^	*U*^13^	*U*^23^
I1	0.01564 (5)	0.01370 (5)	0.02282 (5)	0.00055 (3)	0.00239 (3)	0.00193 (3)
N1	0.0111 (5)	0.0116 (5)	0.0110 (5)	0.0031 (4)	0.0018 (4)	−0.0005 (4)
N2	0.0133 (5)	0.0141 (5)	0.0143 (5)	0.0023 (4)	0.0002 (4)	0.0000 (4)
N3	0.0141 (5)	0.0160 (5)	0.0163 (5)	0.0059 (4)	0.0045 (4)	−0.0015 (4)
N4	0.0177 (5)	0.0148 (5)	0.0206 (6)	0.0041 (4)	0.0067 (4)	0.0059 (4)
C1	0.0107 (5)	0.0151 (6)	0.0152 (6)	0.0047 (5)	0.0003 (4)	−0.0011 (5)
C2	0.0117 (5)	0.0176 (6)	0.0161 (6)	0.0033 (5)	0.0026 (5)	−0.0018 (5)
C3	0.0158 (6)	0.0185 (6)	0.0193 (7)	−0.0002 (5)	0.0023 (5)	0.0010 (5)
C4	0.0252 (7)	0.0141 (6)	0.0179 (7)	0.0020 (5)	−0.0012 (5)	0.0032 (5)
C5	0.0298 (8)	0.0216 (7)	0.0297 (8)	0.0089 (6)	0.0006 (6)	−0.0028 (6)
C6	0.0511 (10)	0.0274 (8)	0.0369 (9)	0.0209 (8)	0.0029 (8)	−0.0043 (7)
C7	0.0643 (12)	0.0164 (7)	0.0289 (9)	0.0120 (8)	−0.0064 (8)	−0.0041 (6)
C8	0.0460 (10)	0.0173 (7)	0.0271 (8)	−0.0042 (7)	−0.0086 (7)	0.0026 (6)
C9	0.0294 (8)	0.0201 (7)	0.0211 (7)	−0.0022 (6)	−0.0016 (6)	0.0055 (6)
C10	0.0134 (6)	0.0195 (6)	0.0181 (6)	0.0047 (5)	−0.0009 (5)	0.0016 (5)
C11	0.0152 (6)	0.0206 (6)	0.0107 (6)	0.0046 (5)	−0.0018 (5)	−0.0016 (5)
C12	0.0203 (6)	0.0235 (7)	0.0140 (6)	0.0081 (5)	0.0004 (5)	0.0009 (5)
C13	0.0275 (7)	0.0244 (7)	0.0145 (7)	0.0059 (6)	−0.0015 (5)	0.0041 (6)
C14	0.0214 (7)	0.0365 (8)	0.0137 (7)	0.0019 (6)	0.0022 (5)	0.0062 (6)
C15	0.0200 (7)	0.0407 (9)	0.0152 (7)	0.0105 (6)	0.0037 (5)	−0.0009 (6)
C16	0.0204 (6)	0.0247 (7)	0.0153 (6)	0.0087 (6)	0.0010 (5)	−0.0017 (5)
C17	0.0135 (6)	0.0127 (6)	0.0132 (6)	0.0049 (5)	0.0018 (4)	−0.0025 (5)
C18	0.0138 (6)	0.0171 (6)	0.0142 (6)	0.0062 (5)	0.0022 (5)	−0.0016 (5)
C19	0.0237 (7)	0.0165 (6)	0.0181 (7)	0.0065 (5)	0.0073 (5)	0.0003 (5)
C20	0.0163 (6)	0.0195 (6)	0.0191 (7)	0.0041 (5)	0.0050 (5)	−0.0021 (5)
C21	0.0222 (7)	0.0247 (7)	0.0218 (7)	0.0083 (6)	0.0056 (5)	0.0008 (6)
C22	0.0269 (7)	0.0364 (8)	0.0186 (7)	0.0054 (7)	0.0069 (6)	0.0008 (6)
C23	0.0370 (9)	0.0298 (8)	0.0230 (8)	−0.0024 (7)	0.0113 (7)	−0.0110 (6)
C24	0.0571 (11)	0.0184 (7)	0.0334 (9)	0.0051 (7)	0.0147 (8)	−0.0069 (7)
C25	0.0434 (9)	0.0204 (7)	0.0217 (7)	0.0072 (7)	0.0089 (6)	−0.0003 (6)
C26	0.0173 (6)	0.0222 (7)	0.0215 (7)	0.0113 (5)	0.0044 (5)	0.0009 (5)
C27	0.0147 (6)	0.0250 (7)	0.0247 (7)	0.0119 (5)	0.0083 (5)	0.0019 (6)
C28	0.0184 (6)	0.0285 (7)	0.0301 (8)	0.0106 (6)	0.0065 (6)	−0.0001 (6)
C29	0.0198 (7)	0.0293 (8)	0.0523 (11)	0.0066 (6)	0.0066 (7)	−0.0057 (8)
C30	0.0229 (8)	0.0280 (8)	0.0748 (14)	0.0103 (7)	0.0246 (8)	0.0124 (9)
C31	0.0328 (8)	0.0428 (10)	0.0499 (10)	0.0236 (8)	0.0281 (8)	0.0233 (8)
C32	0.0241 (7)	0.0355 (8)	0.0269 (8)	0.0168 (6)	0.0111 (6)	0.0060 (6)
C33	0.0136 (5)	0.0108 (5)	0.0134 (6)	0.0016 (5)	0.0022 (4)	−0.0003 (5)
C34	0.0236 (7)	0.0166 (6)	0.0137 (6)	0.0003 (5)	0.0030 (5)	0.0017 (5)
C35	0.0260 (7)	0.0250 (7)	0.0274 (8)	0.0129 (6)	0.0136 (6)	0.0084 (6)
C36	0.0172 (6)	0.0232 (7)	0.0434 (9)	0.0111 (6)	0.0128 (6)	0.0171 (7)
C37	0.0188 (7)	0.0237 (7)	0.0419 (9)	0.0060 (6)	0.0042 (6)	0.0091 (7)
C38	0.0212 (8)	0.0293 (8)	0.0658 (13)	0.0067 (7)	−0.0052 (8)	0.0046 (8)
C39	0.0175 (8)	0.0269 (9)	0.1046 (19)	0.0057 (7)	0.0025 (9)	0.0204 (10)
C40	0.0201 (8)	0.0378 (10)	0.123 (2)	0.0169 (8)	0.0307 (10)	0.0538 (13)
C41	0.0259 (8)	0.0417 (10)	0.0671 (12)	0.0213 (7)	0.0251 (8)	0.0364 (9)
C42	0.0221 (7)	0.0203 (7)	0.0243 (7)	0.0073 (5)	0.0088 (5)	0.0103 (6)
C43	0.0180 (6)	0.0153 (6)	0.0315 (8)	0.0033 (5)	0.0067 (6)	0.0038 (6)
C44	0.0262 (7)	0.0305 (8)	0.0349 (9)	0.0103 (6)	0.0051 (6)	0.0100 (7)
C45	0.0253 (8)	0.0449 (10)	0.0573 (12)	0.0186 (8)	0.0064 (8)	0.0101 (9)
C46	0.0337 (9)	0.0540 (12)	0.0659 (14)	0.0257 (9)	0.0133 (9)	−0.0060 (10)
C47	0.0395 (10)	0.0713 (14)	0.0428 (11)	0.0289 (10)	0.0007 (8)	−0.0234 (10)
C48	0.0241 (7)	0.0432 (10)	0.0357 (9)	0.0161 (7)	−0.0013 (7)	−0.0106 (8)
C49	0.0149 (6)	0.0144 (6)	0.0114 (6)	0.0047 (5)	0.0025 (4)	0.0020 (5)
C50	0.0216 (6)	0.0198 (6)	0.0111 (6)	0.0103 (5)	0.0055 (5)	0.0039 (5)
C51	0.0230 (7)	0.0348 (8)	0.0175 (7)	0.0118 (6)	0.0077 (5)	0.0065 (6)
C52	0.0338 (8)	0.0565 (11)	0.0241 (8)	0.0286 (8)	0.0158 (7)	0.0131 (8)
C53	0.0596 (11)	0.0479 (10)	0.0248 (8)	0.0417 (9)	0.0216 (8)	0.0110 (7)
C54	0.0571 (11)	0.0240 (8)	0.0200 (7)	0.0199 (8)	0.0104 (7)	0.0010 (6)
C55	0.0309 (7)	0.0195 (7)	0.0158 (7)	0.0101 (6)	0.0068 (5)	0.0025 (5)

### Geometric parameters (Å, º)

**Table:**

N1—C33	1.5142 (15)	C24—H24A	0.9500
N1—C17	1.5169 (15)	C25—H25A	0.9500
N1—C1	1.5225 (14)	C26—C27	1.5088 (19)
N1—C49	1.5397 (15)	C26—H26A	0.9900
N2—C2	1.4706 (16)	C26—H26B	0.9900
N2—C3	1.4772 (16)	C27—C32	1.393 (2)
N2—C10	1.4800 (16)	C27—C28	1.393 (2)
N3—C18	1.4740 (16)	C28—C29	1.393 (2)
N3—C19	1.4745 (17)	C28—H28A	0.9500
N3—C26	1.4802 (16)	C29—C30	1.378 (3)
N4—C34	1.4605 (17)	C29—H29A	0.9500
N4—C42	1.4626 (16)	C30—C31	1.386 (3)
N4—C35	1.4663 (17)	C30—H30A	0.9500
C1—C2	1.5305 (17)	C31—C32	1.390 (2)
C1—H1A	0.9900	C31—H31A	0.9500
C1—H1B	0.9900	C32—H32A	0.9500
C2—H2A	0.9900	C33—C34	1.5252 (17)
C2—H2B	0.9900	C33—H33A	0.9900
C3—C4	1.5088 (19)	C33—H33B	0.9900
C3—H3A	0.9900	C34—H34A	0.9900
C3—H3B	0.9900	C34—H34B	0.9900
C4—C9	1.392 (2)	C35—C36	1.504 (2)
C4—C5	1.393 (2)	C35—H35A	0.9900
C5—C6	1.390 (2)	C35—H35B	0.9900
C5—H5A	0.9500	C36—C41	1.390 (2)
C6—C7	1.390 (3)	C36—C37	1.398 (2)
C6—H6A	0.9500	C37—C38	1.383 (2)
C7—C8	1.380 (3)	C37—H37A	0.9500
C7—H7A	0.9500	C38—C39	1.383 (3)
C8—C9	1.390 (2)	C38—H38A	0.9500
C8—H8A	0.9500	C39—C40	1.371 (3)
C9—H9A	0.9500	C39—H39A	0.9500
C10—C11	1.5127 (18)	C40—C41	1.411 (3)
C10—H10A	0.9900	C40—H40A	0.9500
C10—H10B	0.9900	C41—H41A	0.9500
C11—C12	1.3935 (19)	C42—C43	1.5147 (19)
C11—C16	1.3985 (18)	C42—H42A	0.9900
C12—C13	1.390 (2)	C42—H42B	0.9900
C12—H12A	0.9500	C43—C48	1.382 (2)
C13—C14	1.386 (2)	C43—C44	1.391 (2)
C13—H13A	0.9500	C44—C45	1.393 (2)
C14—C15	1.387 (2)	C44—H44A	0.9500
C14—H14A	0.9500	C45—C46	1.378 (3)
C15—C16	1.388 (2)	C45—H45A	0.9500
C15—H15A	0.9500	C46—C47	1.386 (3)
C16—H16A	0.9500	C46—H46A	0.9500
C17—C18	1.5265 (16)	C47—C48	1.388 (2)
C17—H17A	0.9900	C47—H47A	0.9500
C17—H17B	0.9900	C48—H48A	0.9500
C18—H18A	0.9900	C49—C50	1.5064 (17)
C18—H18B	0.9900	C49—H49A	0.9900
C19—C20	1.5101 (19)	C49—H49B	0.9900
C19—H19A	0.9900	C50—C55	1.3955 (19)
C19—H19B	0.9900	C50—C51	1.3990 (19)
C20—C25	1.390 (2)	C51—C52	1.389 (2)
C20—C21	1.3931 (19)	C51—H51A	0.9500
C21—C22	1.391 (2)	C52—C53	1.380 (3)
C21—H21A	0.9500	C52—H52A	0.9500
C22—C23	1.384 (2)	C53—C54	1.384 (3)
C22—H22A	0.9500	C53—H53A	0.9500
C23—C24	1.379 (2)	C54—C55	1.394 (2)
C23—H23A	0.9500	C54—H54A	0.9500
C24—C25	1.394 (2)	C55—H55A	0.9500
			
C33—N1—C17	112.71 (9)	C24—C25—H25A	119.6
C33—N1—C1	108.08 (9)	N3—C26—C27	113.79 (10)
C17—N1—C1	107.80 (9)	N3—C26—H26A	108.8
C33—N1—C49	107.91 (9)	C27—C26—H26A	108.8
C17—N1—C49	108.85 (9)	N3—C26—H26B	108.8
C1—N1—C49	111.53 (9)	C27—C26—H26B	108.8
C2—N2—C3	106.50 (10)	H26A—C26—H26B	107.7
C2—N2—C10	112.13 (10)	C32—C27—C28	118.49 (14)
C3—N2—C10	109.80 (10)	C32—C27—C26	120.88 (13)
C18—N3—C19	111.12 (10)	C28—C27—C26	120.58 (13)
C18—N3—C26	110.13 (10)	C29—C28—C27	120.82 (15)
C19—N3—C26	108.59 (10)	C29—C28—H28A	119.6
C34—N4—C42	113.12 (11)	C27—C28—H28A	119.6
C34—N4—C35	113.16 (11)	C30—C29—C28	120.11 (16)
C42—N4—C35	113.38 (10)	C30—C29—H29A	119.9
N1—C1—C2	115.62 (10)	C28—C29—H29A	119.9
N1—C1—H1A	108.4	C29—C30—C31	119.66 (15)
C2—C1—H1A	108.4	C29—C30—H30A	120.2
N1—C1—H1B	108.4	C31—C30—H30A	120.2
C2—C1—H1B	108.4	C30—C31—C32	120.42 (16)
H1A—C1—H1B	107.4	C30—C31—H31A	119.8
N2—C2—C1	115.67 (10)	C32—C31—H31A	119.8
N2—C2—H2A	108.4	C31—C32—C27	120.49 (15)
C1—C2—H2A	108.4	C31—C32—H32A	119.8
N2—C2—H2B	108.4	C27—C32—H32A	119.8
C1—C2—H2B	108.4	N1—C33—C34	116.02 (10)
H2A—C2—H2B	107.4	N1—C33—H33A	108.3
N2—C3—C4	114.09 (11)	C34—C33—H33A	108.3
N2—C3—H3A	108.7	N1—C33—H33B	108.3
C4—C3—H3A	108.7	C34—C33—H33B	108.3
N2—C3—H3B	108.7	H33A—C33—H33B	107.4
C4—C3—H3B	108.7	N4—C34—C33	106.11 (10)
H3A—C3—H3B	107.6	N4—C34—H34A	110.5
C9—C4—C5	118.61 (14)	C33—C34—H34A	110.5
C9—C4—C3	120.12 (13)	N4—C34—H34B	110.5
C5—C4—C3	121.16 (12)	C33—C34—H34B	110.5
C6—C5—C4	120.75 (15)	H34A—C34—H34B	108.7
C6—C5—H5A	119.6	N4—C35—C36	110.46 (11)
C4—C5—H5A	119.6	N4—C35—H35A	109.6
C5—C6—C7	120.03 (17)	C36—C35—H35A	109.6
C5—C6—H6A	120.0	N4—C35—H35B	109.6
C7—C6—H6A	120.0	C36—C35—H35B	109.6
C8—C7—C6	119.59 (15)	H35A—C35—H35B	108.1
C8—C7—H7A	120.2	C41—C36—C37	118.79 (16)
C6—C7—H7A	120.2	C41—C36—C35	122.16 (16)
C7—C8—C9	120.39 (16)	C37—C36—C35	119.04 (13)
C7—C8—H8A	119.8	C38—C37—C36	121.03 (16)
C9—C8—H8A	119.8	C38—C37—H37A	119.5
C8—C9—C4	120.62 (16)	C36—C37—H37A	119.5
C8—C9—H9A	119.7	C37—C38—C39	120.0 (2)
C4—C9—H9A	119.7	C37—C38—H38A	120.0
N2—C10—C11	114.59 (10)	C39—C38—H38A	120.0
N2—C10—H10A	108.6	C40—C39—C38	119.98 (18)
C11—C10—H10A	108.6	C40—C39—H39A	120.0
N2—C10—H10B	108.6	C38—C39—H39A	120.0
C11—C10—H10B	108.6	C39—C40—C41	120.61 (17)
H10A—C10—H10B	107.6	C39—C40—H40A	119.7
C12—C11—C16	118.26 (13)	C41—C40—H40A	119.7
C12—C11—C10	120.72 (12)	C36—C41—C40	119.58 (19)
C16—C11—C10	120.98 (12)	C36—C41—H41A	120.2
C13—C12—C11	121.05 (13)	C40—C41—H41A	120.2
C13—C12—H12A	119.5	N4—C42—C43	111.18 (11)
C11—C12—H12A	119.5	N4—C42—H42A	109.4
C14—C13—C12	120.16 (13)	C43—C42—H42A	109.4
C14—C13—H13A	119.9	N4—C42—H42B	109.4
C12—C13—H13A	119.9	C43—C42—H42B	109.4
C13—C14—C15	119.39 (13)	H42A—C42—H42B	108.0
C13—C14—H14A	120.3	C48—C43—C44	118.90 (14)
C15—C14—H14A	120.3	C48—C43—C42	119.97 (13)
C16—C15—C14	120.55 (13)	C44—C43—C42	121.06 (14)
C16—C15—H15A	119.7	C43—C44—C45	120.30 (16)
C14—C15—H15A	119.7	C43—C44—H44A	119.8
C15—C16—C11	120.58 (13)	C45—C44—H44A	119.8
C15—C16—H16A	119.7	C46—C45—C44	120.35 (16)
C11—C16—H16A	119.7	C46—C45—H45A	119.8
N1—C17—C18	116.01 (10)	C44—C45—H45A	119.8
N1—C17—H17A	108.3	C45—C46—C47	119.50 (16)
C18—C17—H17A	108.3	C45—C46—H46A	120.3
N1—C17—H17B	108.3	C47—C46—H46A	120.3
C18—C17—H17B	108.3	C46—C47—C48	120.14 (18)
H17A—C17—H17B	107.4	C46—C47—H47A	119.9
N3—C18—C17	107.82 (10)	C48—C47—H47A	119.9
N3—C18—H18A	110.1	C43—C48—C47	120.75 (15)
C17—C18—H18A	110.1	C43—C48—H48A	119.6
N3—C18—H18B	110.1	C47—C48—H48A	119.6
C17—C18—H18B	110.1	C50—C49—N1	114.38 (9)
H18A—C18—H18B	108.5	C50—C49—H49A	108.7
N3—C19—C20	113.15 (11)	N1—C49—H49A	108.7
N3—C19—H19A	108.9	C50—C49—H49B	108.7
C20—C19—H19A	108.9	N1—C49—H49B	108.7
N3—C19—H19B	108.9	H49A—C49—H49B	107.6
C20—C19—H19B	108.9	C55—C50—C51	118.94 (13)
H19A—C19—H19B	107.8	C55—C50—C49	120.55 (12)
C25—C20—C21	118.38 (13)	C51—C50—C49	120.45 (12)
C25—C20—C19	119.85 (12)	C52—C51—C50	120.67 (15)
C21—C20—C19	121.68 (12)	C52—C51—H51A	119.7
C22—C21—C20	120.81 (14)	C50—C51—H51A	119.7
C22—C21—H21A	119.6	C53—C52—C51	119.91 (15)
C20—C21—H21A	119.6	C53—C52—H52A	120.0
C23—C22—C21	120.15 (14)	C51—C52—H52A	120.0
C23—C22—H22A	119.9	C52—C53—C54	120.14 (14)
C21—C22—H22A	119.9	C52—C53—H53A	119.9
C24—C23—C22	119.61 (14)	C54—C53—H53A	119.9
C24—C23—H23A	120.2	C53—C54—C55	120.43 (16)
C22—C23—H23A	120.2	C53—C54—H54A	119.8
C23—C24—C25	120.31 (15)	C55—C54—H54A	119.8
C23—C24—H24A	119.8	C54—C55—C50	119.89 (14)
C25—C24—H24A	119.8	C54—C55—H55A	120.1
C20—C25—C24	120.73 (14)	C50—C55—H55A	120.1
C20—C25—H25A	119.6		
			
C33—N1—C1—C2	−176.81 (10)	C32—C27—C28—C29	1.02 (19)
C17—N1—C1—C2	−54.71 (13)	C26—C27—C28—C29	−176.57 (12)
C49—N1—C1—C2	64.73 (13)	C27—C28—C29—C30	0.2 (2)
C3—N2—C2—C1	−173.21 (10)	C28—C29—C30—C31	−1.2 (2)
C10—N2—C2—C1	66.67 (13)	C29—C30—C31—C32	1.0 (2)
N1—C1—C2—N2	89.86 (13)	C30—C31—C32—C27	0.3 (2)
C2—N2—C3—C4	172.35 (10)	C28—C27—C32—C31	−1.26 (19)
C10—N2—C3—C4	−66.04 (14)	C26—C27—C32—C31	176.32 (12)
N2—C3—C4—C9	138.46 (12)	C17—N1—C33—C34	−55.26 (13)
N2—C3—C4—C5	−45.36 (17)	C1—N1—C33—C34	63.76 (13)
C9—C4—C5—C6	−0.1 (2)	C49—N1—C33—C34	−175.49 (10)
C3—C4—C5—C6	−176.38 (14)	C42—N4—C34—C33	132.65 (11)
C4—C5—C6—C7	0.7 (2)	C35—N4—C34—C33	−96.66 (13)
C5—C6—C7—C8	−0.4 (3)	N1—C33—C34—N4	175.70 (10)
C6—C7—C8—C9	−0.4 (2)	C34—N4—C35—C36	159.68 (11)
C7—C8—C9—C4	1.0 (2)	C42—N4—C35—C36	−69.75 (15)
C5—C4—C9—C8	−0.7 (2)	N4—C35—C36—C41	117.24 (14)
C3—C4—C9—C8	175.57 (13)	N4—C35—C36—C37	−61.53 (16)
C2—N2—C10—C11	−87.12 (13)	C41—C36—C37—C38	0.0 (2)
C3—N2—C10—C11	154.70 (11)	C35—C36—C37—C38	178.78 (13)
N2—C10—C11—C12	127.04 (13)	C36—C37—C38—C39	0.7 (2)
N2—C10—C11—C16	−55.22 (17)	C37—C38—C39—C40	−0.9 (2)
C16—C11—C12—C13	0.27 (19)	C38—C39—C40—C41	0.6 (2)
C10—C11—C12—C13	178.07 (12)	C37—C36—C41—C40	−0.3 (2)
C11—C12—C13—C14	0.3 (2)	C35—C36—C41—C40	−179.06 (13)
C12—C13—C14—C15	−0.5 (2)	C39—C40—C41—C36	0.0 (2)
C13—C14—C15—C16	0.0 (2)	C34—N4—C42—C43	−75.77 (15)
C14—C15—C16—C11	0.6 (2)	C35—N4—C42—C43	153.65 (12)
C12—C11—C16—C15	−0.71 (19)	N4—C42—C43—C48	−44.20 (18)
C10—C11—C16—C15	−178.50 (12)	N4—C42—C43—C44	138.73 (14)
C33—N1—C17—C18	−56.95 (13)	C48—C43—C44—C45	−1.9 (2)
C1—N1—C17—C18	−176.14 (10)	C42—C43—C44—C45	175.18 (14)
C49—N1—C17—C18	62.73 (13)	C43—C44—C45—C46	0.2 (3)
C19—N3—C18—C17	69.30 (13)	C44—C45—C46—C47	1.7 (3)
C26—N3—C18—C17	−170.33 (10)	C45—C46—C47—C48	−1.9 (3)
N1—C17—C18—N3	162.61 (10)	C44—C43—C48—C47	1.7 (3)
C18—N3—C19—C20	−163.59 (10)	C42—C43—C48—C47	−175.40 (17)
C26—N3—C19—C20	75.13 (13)	C46—C47—C48—C43	0.2 (3)
N3—C19—C20—C25	−144.93 (13)	C33—N1—C49—C50	−68.30 (12)
N3—C19—C20—C21	38.60 (17)	C17—N1—C49—C50	169.07 (10)
C25—C20—C21—C22	−0.6 (2)	C1—N1—C49—C50	50.26 (13)
C19—C20—C21—C22	175.93 (13)	N1—C49—C50—C55	92.30 (14)
C20—C21—C22—C23	0.2 (2)	N1—C49—C50—C51	−90.69 (14)
C21—C22—C23—C24	0.5 (2)	C55—C50—C51—C52	−1.3 (2)
C22—C23—C24—C25	−0.7 (3)	C49—C50—C51—C52	−178.31 (12)
C21—C20—C25—C24	0.3 (2)	C50—C51—C52—C53	1.4 (2)
C19—C20—C25—C24	−176.25 (15)	C51—C52—C53—C54	−0.8 (2)
C23—C24—C25—C20	0.3 (3)	C52—C53—C54—C55	0.1 (2)
C18—N3—C26—C27	67.15 (14)	C53—C54—C55—C50	0.0 (2)
C19—N3—C26—C27	−170.96 (11)	C51—C50—C55—C54	0.55 (19)
N3—C26—C27—C32	70.99 (16)	C49—C50—C55—C54	177.60 (12)
N3—C26—C27—C28	−111.48 (13)		

### Hydrogen-bond geometry (Å, º)

**Table:**

*D*—H···*A*	*D*—H	H···*A*	*D*···*A*	*D*—H···*A*
C39—H39*A*···I1^i^	0.95	3.01	3.893 (2)	154
C49—H49*B*···I1^ii^	0.99	2.86	3.8187 (13)	162

Symmetry codes: (i) −*x*, −*y*, −*z*+1; (ii) −*x*+1, −*y*+1, −*z*+1.
